# Primary Mediastinal B-Cell Lymphoma: Novel Precision Therapies and Future Directions

**DOI:** 10.3389/fonc.2021.654854

**Published:** 2021-03-22

**Authors:** Huan Chen, Tao Pan, Yizi He, Ruolan Zeng, Yajun Li, Liming Yi, Hui Zang, Siwei Chen, Qintong Duan, Ling Xiao, Hui Zhou

**Affiliations:** ^1^ Department of Lymphoma and Hematology, Hunan Cancer Hospital, The Affiliated Cancer Hospital of Xiangya School of Medicine, Central South University, Changsha, China; ^2^ The Third Xiangya Hospital, Central South University, Changsha, China; ^3^ Department of Human Anatomy, Hunan University of Medicine, Huaihua, China; ^4^ Department of Basic Medicine, Yiyang Medical College, Yiyang, China; ^5^ Department of Histology and Embryology of School of Basic Medical Science, Central South University, Changsha, China

**Keywords:** primary mediastinal large B-cell lymphoma, chemotherapy, targeted therapy, mediastinal radiation, positron emission tomography-computed tomography

## Abstract

Primary mediastinal large B-cell lymphoma (PMBCL) is a distinct clinicopathologic disease from other types of diffuse large B-cell lymphoma (DLBCL) with unique prognostic features and limited availability of clinical data. The current standard treatment for newly diagnosed PMBCL has long been dependent on a dose-intensive, dose-adjusted multi-agent chemotherapy regimen of rituximab plus etoposide, prednisone, vincristine, cyclophosphamide, and doxorubicin (DA-R-EPOCH). Recent randomized trials have provided evidence that R-CHOP followed by consolidation radiotherapy (RT) is a valuable alternative option to first-line treatment. For recurrent/refractory PMBCL (rrPMBCL), new drugs such as pembrolizumab and CAR-T cell therapy have proven to be effective in a few studies. Positron emission tomography-computed tomography (PET-CT) is the preferred imaging modality of choice for the initial phase of lymphoma treatment and to assess response to treatment. In the future, baseline quantitative PET-CT can be used to predict prognosis in PMBCL. This review focuses on the pathology of PMBCL, underlying molecular basis, treatment options, radiotherapy, targeted therapies, and the potential role of PET-CT to guide treatment choices in this disease.

## Introduction

Primary mediastinal large B-cell lymphoma (PMBCL) is a rare subtype of non-Hodgkin lymphoma (NHL), but it shares histologic features with nodular sclerosing Hodgkin lymphoma (NSHL) (1, 2). Due to the distinct clinical, morphological, and immunophenotypic characteristics, it is recognized as a unique entity in the latest World Health Organization classification of lymphoid tumors (3). Optimal treatment is not fully defined and there is no single standard of care. However, several retrospective studies have found that patients with PMBCL have a high survival rate with chemotherapy using dose-adjusted multi-agent chemotherapy regimen of rituximab plus etoposide, prednisone, vincristine, cyclophosphamide, and doxorubicin (DA-R-EPOCH) or a combination of rituximab and CHOP (cyclophosphamide, doxorubicin, vincristine, prednisone [R-CHOP]) (4). The survival rate of PMBCL is significantly higher than diffuse large B-cell lymphoma (DLBCL) (5). Over the past 20 years, several studies have been carried out to understand its clinical behavior and optimize its management.

## Clinical Features

PMBCL accounts for 2% to 4% of all NHL. The incidence of the disease in women is higher than in men (4, 6, 7). In more than two-thirds of cases, it appears as a large anterior mediastinal mass, often accompanied by superior vena cava syndrome and airway obstruction (8). Approximately one-third of patients present B symptoms (fever, weight loss, night sweats). Additional, clinical features consist of pleural and pericardial effusions, elevated serum lactate dehydrogenase (LDH) concentrations, and a trend toward recurrence at uncommon sites. Patients often present with cough and bout a quarter of patients present with advanced disease (9). The primary appearance can be nodular or extranodal; however, recurrences are often extranodal and may involve the gastrointestinal tract, liver, kidneys, and ovaries (10). Bone marrow involvement has been observed in only 1–5% of cases. Central nervous system (CNS) involvement in the form of leptomeningeal or intraparenchymal disease is uncommon and is especially seen in extranodal disease and in recurrences  (11, 12). In addition to the above common symptoms, chylous pleural and pericardial effusions are present in some children (13). Moreover, PMBCL cases have been found in non-mediastinal areas, which makes the correct diagnosis of PMBCL difficult (14, 15). The poor prognostic characteristics of patients treated with R-CHOP include higher International Prognostic Index (IPI) score, advanced stage, advanced age, and multiple extranodal sites (16, 17).

## Immunohistochemistry Features

PMBCL is an aggressive B-cell lymphoma that arises in the mediastinum and has a putative thymic B-cell origin. Tumor cells have an immunophenotype compatible with B-cell characteristics, such as the positive expression of CD20, CD45, CD79a, while the expression of cellular immunoglobulins, HLA I/II, CD5, CD3, CD21, and CD10 is negative. Furthermore, CD30 expression is observed in over 80% of PMBCL, although it is weaker and more heterogeneous than that observed in classical Hodgkin Lymphoma (cHL) (18–20). Tumor cells are usually positive for nuclear transcription regulators such as BOB1, PU.1, OCT2, PAX5, BCL6, and IRF4. Bcl-6 protein has been detected in over 50% of tumor cells and is considered a favorable prognostic factor (21, 22) (**Table 1**).

**Table 1:** Comparison of PMBCL with other mediastinal lymphoma diseases in pathological diagnosis.

	cHL	PMBCL	DLBCL
Morphological features	Lacunar Hodgkin Reed-Sternberg cells; Epithelioid granuloma.	Sheets of large cells; no inflammatory; reticulated or alveolar patterns of fibrosis	Sheets of large cells with variable aspects
Immunophenotypic features	CD30 positive. CD15 usually expressed. Transcription factors OCT2 and BOB1 usually not expressed, PAX5 is weak or negative.	Strong expression of B-cell antigens, such as CD20. Weak and variable CD30 expression. 70% express CD23, MAL, PDL-1 and PDL-2. Absent surface immunoglobulin. Transcription factors PAX5, OCT2, BOB1 expressed.	Strong expression of B-cell antigens such as CD20. Surface immunoglobulin typically present. Transcription factors PAX5, OCT2, BOB1 usually expressed. CD30 rarely expressed.
Genetic features	Chromosome alterations: 9p24.1/CD274/PDCD1LG2; Mutations: B2M, TNFAIP3, PTPN1.	Chromosome alterations: 9p24.1/CD274/PDCD1LG2; Mutations: JAK/STAT, NF-ĸB, CIITA, CD58, B2M, CD274, PDCD1LG2, ITPKB, MFHAS1, XPO1, IRF2BP2, IRF4, IRF8; Amplifications: TRAF1.REL, BCL10, MALT1; Inactivation: TNFAIP3	Mutations: CD79a/b, MYD88, NOTCH1, NOTCH2, NF-ĸB, BCL10, TNFAIP3, EZH2; Rearrangement: MYC, BCL-2 and/or BCL-6; Amplifications: REL; Inactivation: TNFRSF14, CREBBP, and EP300.

## Genetic Features

PMBCL has unique genetic characteristics that affect the biological behavior of tumors. Studies defining the gene-expression profiles of PMBCL could help predict the prognosis of patients. However, sometimes it is difficult to make the final diagnosis of PMBCL, as it may overlap with other types of lymphoma. Compared with DLBCL, the identified driver genes were found to be significantly more frequently mutated in PMBCL, while only a restricted number of genes were significantly different between PMBCL and cHL (23–25). Recent studies have established that PMBCL pathobiology is reliant on molecular pathways involving REL, JAK-STAT, PD-L1/PD-L2, and Nuclear factor-κB (NFκB) (23). Through comprehensive genomic analysis, Mottok et al. found that highly duplicated oncogenic mutations in genes belonging to the JAK-STAT and NF-kB pathways (CIITA, CD58, B2M, CD274, PDCD1LG2) were closely associated with immune evasion (25). This study also highlighted the frequent mutations in ITPKB, MFHAS1, XPO1, and NFKBIE in PMBCL, and also found that multiple members of the pathway were frequently mutated in the IRF pathway (IRF2BP2, IRF4, IRF8) (25–27). Hao et al. found that disease-specific chromosome 9p24.1/JAK2 amplification increased JAK2 expression and activity in PMBCL (28). DD1 et al. reported that the programmed death ligand-1 (PD-L1) locus (9p24.1) was frequently and specifically rearranged in PMBCL (20%) when compared with DLBCL, follicular lymphoma (FL), and Hodgkin lymphoma (HL) among 571 different B-cell lymphomas samples (29). Gene expression profiling studies showed that tumor necrosis factor (TNF) family members and TRAF1 are overexpressed in PMBCL (23). In PMBCL, this overactivation leads to activation of downstream anti-apoptotic genes, activation of caspases, and transcription of cell cycle regulators, resulting in malignant proliferation (30). The two most common genetic changes in PMBCL are CIITA rearrangement and chromosome amplification of 9p24.1 (PDL1/PDL2) and 2p14 p16 (24, 31, 32). Steidl et al. found that the presence of CIITA rearrangement was significantly associated with shorter disease-specific survival rates (33, 34). Relapsing somatic mutations in the NF-kB and JAK-STAT6 signaling pathways lead to their abnormal activation and constituted a hallmark of the disease. Somatic IL4R mutations in PMBCL resulted in constitutive activation of JAK-STAT signaling (35). In fact, gene expression profiles of characteristic genes in HL and PMBCL indicated that PMBCL is one-third identical to NSHL. These characteristic genes are not expressed in other types of DLBCL, but high expression of PDL2 is common to both PMBCL and HL (24). Chapuy et al. analyzed the genomes of 37 newly diagnosed PMBCL patients. They identified nine major genetic drivers in each PMBCL patient. The most obvious mutant gene in this study was B2M, which encodes β2-microglobulin, and is responsible for the correct translocation of MHC class I molecules to the intracellular plasma membrane and is essential for the expression of endogenously degraded autoantigens and non-autoantigens (36, 37). In addition, ZNF217 mutations are often present in patients with PMBCL. These newly identified mutations may increase susceptibility to programmed cell death-1 (PD-1) receptor blockade, including high tumor mutational load, microsatellite instability, and an APOBEC mutational signature (36).

**Figure 1:**
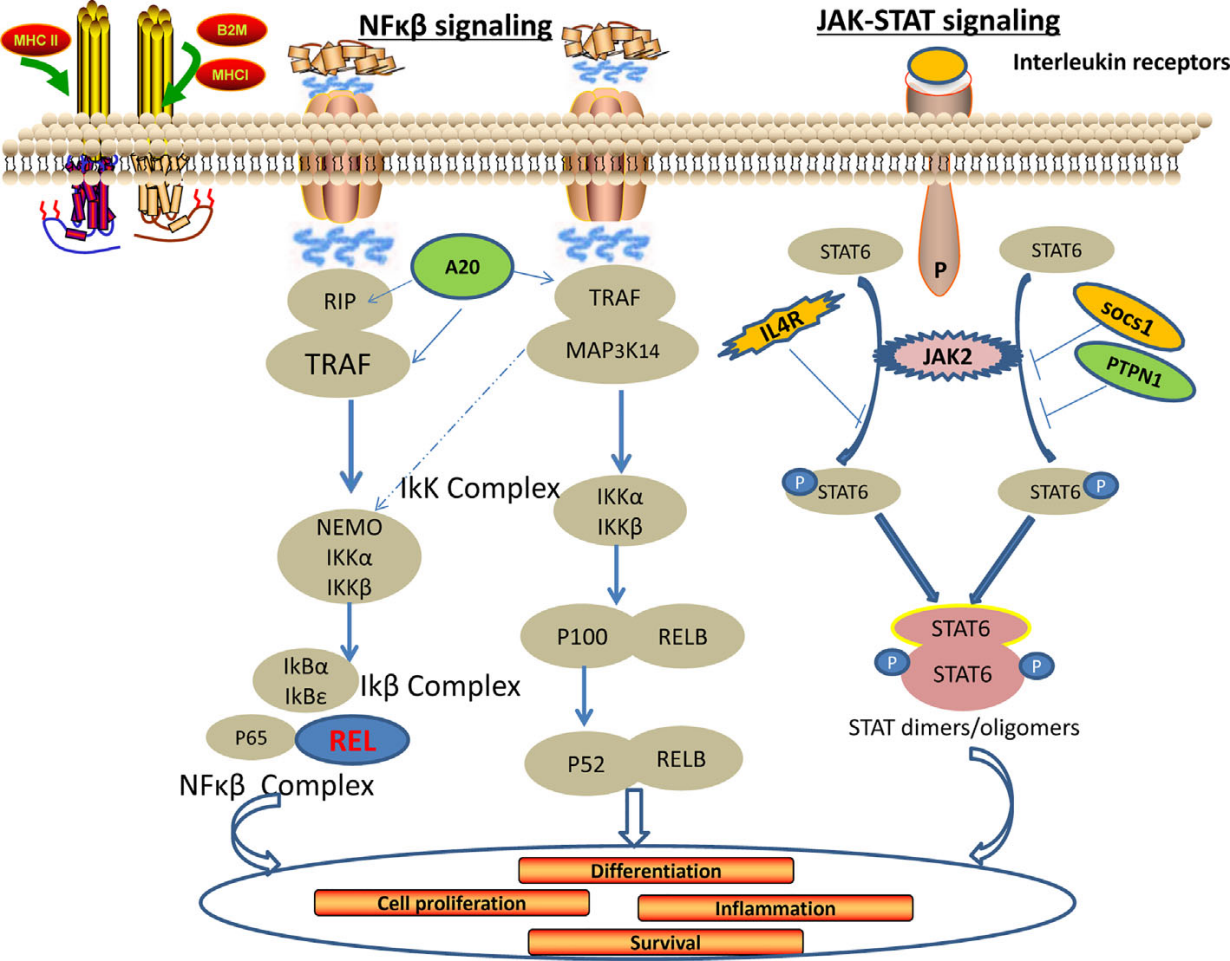
A popular signaling pathway in the research of primary mediastinal large B-cell lymphoma.

The use of gene expression profiles can develop more precise molecular diagnostics for PMBCL. Studies have identified molecular pathways of PMBCL dependent on REL, JAK-STAT, PD-L1/PD-L2, and NF-κB (38). These molecular pathways may become targets for rational therapeutic strategies for PMBCL (**Figure 1**).

## Role of Imaging in PMBCL

The prognosis of most patients with PMBCL is very impressive as most patients respond to treatment, but some patients still present substantial sclerosis in the mediastinal after treatment, which is translated after therapy into the presence of residual tumor masses in most cases. Ordinary imaging studies such as computed tomography (CT) and X-ray imaging are unable to recognize residual lesions. Positron emission tomography-computed tomography using 2-deoxy-2-[18F] fluoro-D-glucose (PET-CT) can be used to distinguish between benign and malignant tumors. Therefore, when selecting tumor biopsy sites and staging, PET-CT is recommended over CT (39). According to Ujjani et al. PET-CT was highly accurate for detecting bone marrow involvement (BMI) at diagnosis in DLBCL and HL, and highly specific in FL at diagnosis and relapse. It can even detect BMI in patients with negative biopsy (40). Furthermore, RHM et al. investigated 56 patients and reported being able to discriminate between mediastinal Hodgkin’s disease and PMBCL by combining LDH levels with PET-CT findings (41).

In general, PET-CT imaging plays a central role in the disease staging and response assessment of lymphoma patients and has become an important part of successful treatment management strategies (39, 42). To avoid the lack of objectivity in the results of PET-CT, a five-point scale (Deauville criteria) was designed to assess the patient’s condition. The International Conference on Malignant Lymphomas Imaging Working Group considers negative PET-CT (Deauville scores 1–3) may represent a complete response (CR) in a lymphoma patient treated with standard therapy (43, 44).

The prospective IELSG-26 study reported using liver uptake as the cutoff for PET-CT positivity (the boundary of a score, 3 to 4) to distinguish between high failure risk and low failure risk, with 5-year progression-free survival (PFS) of 99% versus 68% (P<0.001) and the 5-year overall survival rate (OS) of 100% versus 83% (P<0.001) (45). The PET-CT scan findings of 88 patients with PMBCL who received immunochemotherapy and radiation therapy (RT) were reported in the IELSG-26 study. Patients with a complete metabolic response (Deauville score 3) did not progress after 5 years, confirming the value of the Lugano classification criteria in the assessment of PMBCL response after RT. After combining RT, the complete metabolic response (CMR) (Deauville score < 4) can be substantially increased (from 74% to 89%). Therefore, PET-CT can identify patients with a high risk of progression after RT (46). The PETAL trial reported that two doses of rituximab following six cycles of R-CHOP did not improve outcomes, according to PET-CT results (47). Several studies have proved the Deauville score of PET-CT can be used to guide the subsequent treatment of PMBCL, and even predict the survival rate after chemoimmunotherapy for PMBCL (45, 48, 49). The International Study Group of Extranodal Lymphoma (IELSG37) study found that the Deauville scoring standard was used, and the final PET-CT scan results were more consistent, which was also recognized by the Lugano classification standard (50). An observational retrospective single-center study reported the experience of PMBCL patients treated with the third-generation chemotherapy regimen R-MACOP-B (rituximab, methotrexate, doxorubicin, cyclophosphamide, vincristine, prednisone, and bleomycin). After treatment with R-MACOP-B, 82.4% of the patients achieved CR and the 10-years OS was 82%. The PFS rate was 87.6% and the disease-free survival rate (DFS) was 90.5% (median follow-up 4 years). There was no statistically significant difference in the DFS between the two groups in the final observation group (PET-negative) for patients with PET-positive RT consolidation treatment: 90.7% and 90%, respectively (p = 0.85). The study’s results indicated that PET-CT could be used as a guide for patients to undergo RT consolidation therapy in the later disease stages, and it is also convenient to reduce the use of RT. Due to a lack of long-term follow-up data and prospective head-to-head trials, the study could not firmly establish the best treatment plan (51). Nonetheless, treatments must strike a balance between maximizing cure rates and minimizing long-term toxicity (52).

A recent study of 159 PMBCL patients with 94% of them receiving R-CHOP treatment showed that the 5-year time to progression (TTP) and OS of the entire cohort was 80% and 89%, respectively. A total of 113 patients underwent PET-CT scans: 63% were negative and 37% were positive, the 5-year TTP was 90% vs. 71% and the 5-year OS was 97% vs. 88%, respectively. For patients using the Deauville score PET-CT scan (n = 103), the percentage of PET-negative cases (Deauville score 1–3) was 91%. Moreover, the difference in 5-year TTP prognosis between Deauville score 4 and Deauville score 5, was 33% and 87%, respectively, P = 0.0002). This study clearly demonstrated that PMBCL patients could achieve considerable curative effects through the R-CHOP regimen, and using PET-CT adaptive therapy could reduce exposure to RT for most PET-negative patients (53). A limited number of patients Deauville score 4 in IELSG-26 study also achieved satisfactory outcomes (46). Filippi et al. performed a series of combined treatments on 51 patients with PMBCL and obtained similar results. The results showed that 17 patients with Deauville score 4 posterior branch had a good prognosis and no recurrence (54). These false-positive results were thought to be due to residual inflammation thymic rebound after mediastinal treatment or residual 18f-fluoro-D-glucose uptake may not have reflected persistent lymphoma (45, 46).

Despite the possibility of PET-CT substantially increasing the specificity, of diagnosis, there is the potential of false positivity in predicting prognostic. Newer studies are enhancing the prognosis value of PET-CT. For example, PET-CT baseline quantitative parameters, the maximum normalized uptake value (SUVmax), metabolic tumor volume (MTV), and total lesion glycolysis (TLG) are powerful predictors of PMBCL prognosis (55–57). Ceriani et al. determined that functional PET-CT parameters such as TLG could be used to predict patient outcomes. On multivariate analysis, only TLG retained a significant association with OS (P = 0.01) and PFS (P < 0.01). At 5 years, the OS of patients with low TLG was 100%, while the OS of patients with high TLG was 80% (P = 0.001), while the PFS was 99% and 64%, respectively (P < 0.001) (58). Similar results can be reflected in the study by Pinnix et al. A retrospective analysis was performed in 65 newly diagnosed patients with PMBCL. These patients were evaluated by PET-CT during first-line treatment with DA-R-EPOCH. Evaluation factors included evaluation of MTV and TLG. The median follow-up time was 36.6 months (95% confidence interval [CI] = 28.1–45.1). The 2-year PFS and OS rates of these 65 patients were 81.4% and 98.4%, respectively. The statistical analysis showed that the baseline MTV and TLG thresholds were associated with poor PFS. However, other pretreatment clinical factors, including the IPI and large tumor mass (> 10 cm) disease, were not statistically different (56). In the multivariate analysis, only TLG retained statistical significance (P = 0.049). Univariate analysis of post-treatment variables showed that residual CT tumor volume, maximum standardized uptake value, and the Deauville score were related to PFS. In the multivariate analysis, the Deauville score of 5 was still significant (P =0.006). A model that combines the evaluation of baseline TLG and the Deauville score at the end of treatment could identify patients with a high risk of progression (56, 57). Therefore, this prognostic model may screen high-risk patients for more intensive treatment or even combine with new targeted treatments such as pembrolizumab (53). In the future, baseline quantitative PET-CT may be used to provide an earlier definition of a risk-adapted therapeutic strategy in PMBCL through this new tumor metabolizing biomarker.

## Frontline Treatment

The treatment of this PMBCL remains an area of active research. Previously, most clinical management to support clinical care was inferred from retrospective studies. Before the rituximab-era, some retrospective studies suggested that outcomes of patients with V/MACOP-B (etoposide, doxorubicin, cyclophosphamide, vincristine, prednisone, bleomycin) were superior to CHOP (5, 9, 59, 60). A retrospective multicenter report in Italy showed a 51.1% reduction in CR in the CHOP group and an 80% reduction in the V/MACOP-B group (P < 0.001). The recurrence rate was 22.7% in patients receiving CHOP and 9.2% in patients receiving V/MACOP-B. CHOP was 39.5% in patients without events and 75.7% in the V/MACOP-B group (P < 0.001) (59). Historically, some European centers have strongly supported the above view, although the efficacy of CHOP was relatively poor (61, 62). However, the CHOP regimen is more common for the treatment of PMBCL in the United States of America, possibly due to the early classification of PMBCL into the DLBCL subtype (63, 64).

Since adding rituximab to CHOP, the benefits of V/MACOP-B are no longer clear (65). Savage et al. reported the 5-year OS rate in patients aged < 65 years old treated with V/MACOP-B and R-CHOP was 87% and 81%, respectively. In a pair-wise survival comparison, there was no significant difference between the V/MACOP-B and R-CHOP regimens. A retrospective study compared 45 patients who received the V/MACOPB-plus-rituximab regimen with mediastinal radiotherapy. There were no statistical differences in CR and relapse-free survival (RFS) when compared with historical data for rituximab-free V/MACOP-B (66). However, cure rates improved dramatically with CHOP plus rituximab. The purpose of the Rituximab International Trial Group was to evaluate the effects of CHOP in combination with rituximab on PMBCL; the authors concluded that rituximab increased the CR rate of PMBCL (67). At the same time, another retrospective study confirmed satisfactory results of R-CHOP (16, 65, 68).

Tai et al. reported the OS and PFS for R-CHOP and CHOP treated patients were 87% vs. 57% and 88% vs. 36%, respectively (after a median follow-up of 31.2 months) (69). Conversely, Soumverain et al. raised the concern that the incidence of primary refractory diseases in PMBCL patients treated with R-CHOP was unacceptably high, particularly for patients with advanced-stage or high IPI risk scores, and thus R-CHOP appeared to be inadequate for chemotherapy. Low-risk patients receiving R-CHOP may require RT consolidation at a later stage (16). A recent long-term retrospective study that investigated the clinical effects of a 12-cycle V/MACOP-B regimen with or without rituximab in 151 PMBCL patients, 120 patients (79.5%) achieved a CR, and 12 patients (7.9%) a partial remission (Objective Response Rate ORR: 87.4%). The 21-year OS was 82.6%; the PFS and DFS rates were 69.3% and 86.4%, respectively. This study also presented long-term follow-up data, indicating that third-generation chemotherapy such as MACOP-B is feasible in the treatment of PMBCL (70).

In 2013, the NCI conducted a single-arm, phase II, prospective study of 51 patients with untreated PMBCL and without combined radiotherapy. The Mean follow-up of 5 years revealed 93% event-free survival and 97% OS (71). Based on these results, this regimen has been considered a standard of care by many centers around the world. However, there is still a lack of prospective studies to verify these results. Similarly, Wilson et al. agreed that DA-R-EPOCH chemotherapy has high efficacy in previously untreated B-cell lymphomas (72, 73). Some studies have shown that R-CHOP may replace DA-R-EPOCH in the treatment of PMBCL patients. Although the CR rate of DA-R-EPOCH is higher than that of R-CHOP (84% vs. 70%, P = 0.046), patients receiving DA-R-EPOCH were more likely to experience treatment-related toxicity. The 2-year OS of 89% versus 91%, indicated that there was no significant difference between these two chemotherapy regimens (74). It has also been observed that grade I–II cardiac complications in the DA-R-EPOCH group were more frequent. Another study including 53 cases of PMBCL treated with R-CHOP (n = 21) and DA-R-EPOCH (n = 28), indicated there was no difference in the 1-year PFS and OS between the two groups (17, 75). In conclusion, treatments for PMBCL have evolved over time. Although, patients who receive the DA-R-EPOCH regimen are more likely to experience short-term toxic side effects (17).

The early, lymphoma tumor group recommended combined therapy consisting of RT followed 6 cycles of CHOP-type chemotherapy. Some previous reports have indicated that consolidation RT induces a good remission rate. The National Cancer Database suggested superior 5-year OS for the no-RT and RT groups, 83% versus 93%, respectively (76). However, a retrospective review failed to show any additional benefit in the RT group with regard to PFS or OS among the 45 patients treated with CHOP and R-CHOP (38). Similar results were found in the Savage et al. report, whereby the conventional addition of radiotherapy did not improve survival (5). Further, Messmera et al. reported there was no significant difference in PFS or OS between the R-CHOP group and the R-CHOP plus radiation group (74).

**Figure 2:**
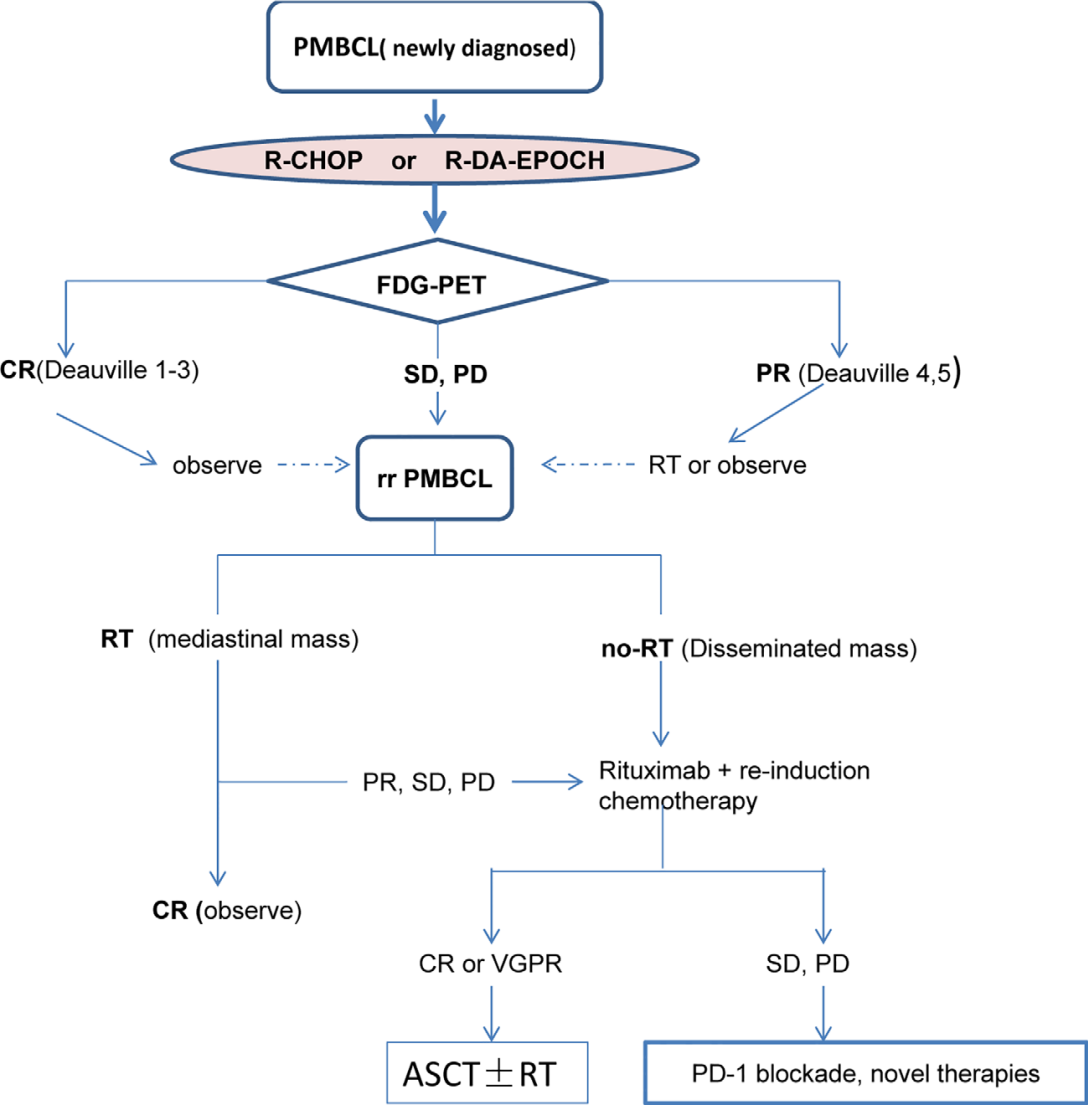
The current routine procedure of clinical treatment of PMBCL.

Given the good results achieved by the incorporation of rituximab and dose-enhanced chemotherapy, RT may be avoidable in most patients (52, 71, 77). A single-center retrospective study advanced the proposal that therapy with DA-R-EPOCH could avoid the need for radiotherapy in PMBCL (71). Most patients who received dose-enhanced chemotherapy could abandon consolidative mediastinal RT without any compromise in long-term outcomes. Similar results were obtained by Malenda et al. study (71, 75, 78). More recently, Jiang et al. and others have made alternative proposals. They analyzed 474 patients with PMBCL, which included 65.8% of patients aged 18–39 years old and 34.2% of patients aged 40–59 years old; of these 45.8% received RT. Univariate analysis revealed that exposure to RT was associated with prognosis in patients aged 40–59 years (after adjusting for tumor stage and race). However, RT treatment could not be administered to patients aged 18–39 years (79). In addition, Chan et al. reported a 5-year PFS of 88% in PMBCL patients receiving either R-CHOP (n = 41), R-CHOP + RT (n = 37) or DA-R-EPOCH (n = 46); a minority of patients in the DA-R-EPOCH arm received radiation. PFS was superior in patients treated with R-CHOP + RT or DA-R-EPOCH than in patients treated with R-CHOP alone, with the 5-year PFS of 90% versus 88.5% versus 56%, respectively (P = 0.02). These findings indicated that both R-CHOP+RT and DA-R-EPOCH could provide an excellent prognosis for PMBCL patients. In particular, patients receiving R-CHOP treatment, especially in those patients with larger disease size, non-radiotherapy consolidation treatment led to poorer PFS (80). In addition, Use of RT in PMBCL may predispose young patients to cardiopulmonary toxicity and secondary malignancies (81). RT therapy for PMBCL is a subject of ongoing debate, with no accepted standard of care. It is undeniable that consolidation RT is particularly valuable for patients with residual disease (Deauville score > 3), as RT can convert a partial response (PR) into full response CR after chemotherapy (59, 62). However, it is still uncertain whether CR patients need to follow-up with RT or not. Therefore, further studies are needed to establish the precise role of RT. The disadvantage of the R-CHOP or MACOP sequential RT regimen is that RT increases the patient’s medical expenses and also increases the risk of secondary tumors (82). Currently, there is no universally accepted standard of care for the initial treatment of PMBCL and there have been only retrospective analyses of data that we will discuss below (**Figure 2**; **Table 2**).

**Table 2:** Results of chemotherapy data for primary mediastinal large B-cell lymphoma reported in various studies.

Study	Year	Number of patients	chemotherapy regimens	RT	Survival	Outcome
Zinzani et al. (61)	1996	22	MACOP-B/F-MACHOP	100%	87% (2-y-PFS)	Good results with third generation regimens plus radiotherapy
Savage et al. (5)	2006	47 18 63	MACOP-B/VACOP-B CHOP-R CHOP type		87% 82% (5y-OS) 71%	CHOP is less effective than MACOP-B/VACOP-B Rituximab improves curative effect
Zinzani et al. (66)	2009	45	R-V/MACOP-B	71%	80% (5y-OS)	There are no statistically significant differences between R-V/MACOP-B and V/MACOP-B
Messmera et al. (17)	2019	43	CHOP-R	23%	93% (3y-OS) 100%(3y-PFS)	R-CHOP alone has a high cure rate in PMBCL
Rieger et al. (67)	2011	44 43	R-CHOP-like CHOP-like	70%	78% (3yr-EFS) 52% (3yr-EFS)	Rituximab combined with CHOP chemotherapy is an effective treatment for PMBCL with a good prognosis
Vassilakopoulos et al. (68)	2012	66 29	R-CHOP CHOP	79% 48%	96% (5y-OS) 86% (5y-OS)	Whether or not combined with RT, PMBCL has a good effect on R-CHOP chemotherapy.
Dunleavy et al. (71)	2013	51	DA-EPOCH-R	0	97% (5y-OS) 93% (5y-PFS)	DA-EPOCH-R treatment can avoid the need for radiotherapy in patients with PMBCL.
Giulino-Roth et al. (77)	2017	118	DA-EPOCH-R	87%	95% (3y-OS) 87% (3y-EFS)	DA-R-EPOCH regimen has a good effect in PMBCL and support the use of DA-EPOCH-R for the treatment of PMBCL in children and adults.

## Relapsed/Refractory Setting

Despite the high efficacy of immunochemotherapy regimens, a minority of patients with PMBCL experience relapsed/refractory disease. The prognosis among cases of rrPMBCL is poor (59, 83). The standard treatment for rrPMBCL is salvage therapy followed by hematopoietic stem cell transplantation (HSCT). A report by the European Society of Blood and Bone Marrow Transplantation in PMBCL sensitive to chemotherapy indicated that autogenic stem-cell transplantation (auto-SCT) with or without RT exhibits a good prognosis, while, its benefits seem to be limited in patients with rrPMBCL (84). Another multicenter study reported the results of allogeneic transplantation in patients with rrPMBC (n = 28). Approximately 79% of patients were sensitive to high-dose chemotherapy before transplantation and in this population, the 5-year PFS, 5-year OS, and non-recurring population, and the cumulative incidence of death and recurrence were 34%, 45%, 32%, and 33%, respectively. Compared with refractory patients with a 2-year PFS and OS of 0% each, the outcome of patients was significantly better. Nonetheless, the major morbidity and mortality risks cannot be ignored and the associated high medical costs are not conducive to popularizing this approach (85). The pathobiological and genetic characteristics of PMBCL include motivate and contribute to the hope for the development of new therapeutic drugs for rrPMBCL in the future.

### Novel Agents for PMBCL

PMBCL is associated with 9p24 genetic abnormalities and overexpression of PD-1 ligand (PD-L1), thus it has been speculated that PMBCL may be susceptible to PD-1 blockade (28, 29, 32). Pembrolizumab is an effective and well-tolerated treatment option that has been approved for use in a variety of cancer types, from advanced melanoma to relapsed/refractory cHL (rr-cHL) (86, 87). Several agencies are currently evaluating the role of pembrolizumab in the treatment of rrPMBCL. There are phase II trials of pembrolizumab in rrPMBCL currently ongoing (KEYNOTE-013/KEYNOTE-170). An interim assessment of this trial reported that the ORR was 48% (7 complete responses; 33%) among the 21 patients in KEYNOTE-013 and 45% (7 complete responses; 13%) among the 53 patients in KEYNOTE-170. Pembrolizumab was also reported to be well tolerated, with treatment-related adverse events in most patients being of low grade. There have been no treatment-related deaths reported to date. Pembrolizumab has exhibited a high response rate, long-lasting activity, and manageable safety in patients with rrPMBCL (88, 89).

As mentioned above, another potential therapeutic target for PMBCL is the JAK/STAT signaling pathway (90). Both the JAK2 inhibitor ruxolitinib and the JAK2/FLT3 inhibitor SB518 have been evaluated in HL and PMBCL (91). However, due to the currently small number of cases evaluated, their efficacy cannot be definitely determined.

Brentuximab vedotin (BV) is an antibody-drug conjugate targeting the CD30 antigen, which is highly expressed in cHL and in systemic anaplastic large cell lymphomas (sALCL). In 2012, the Food and Drug Administration (FDA) approved the use of BV in cHL and sALCL (92). Other studies have confirmed the efficacy of BV in patients expressing high levels of CD30 (93–95). PMBCL is also characterized by an elevated expression of CD30 (20). Therefore, a single-arm phase II trial was conducted to observe the use of BV in patients with rrPMBCL. In this study, the expression of CD30 (usually weak in PMBCL) was not associated with response rates. The mid-term evaluation showed that the ORR was unexpectedly low and the study was terminated prematurely (88). Recently, a multi-center research study reported that the combination of nivolumab (anti-PD-1 checkpoint inhibitor) and BV may exert synergistic activity in rrPMBCL. Among 30 patients treated, ORR (95% CI) was 73%, with a 37% CR rate per investigator, and an ORR of 70%, with a 43% complete metabolic response rate per independent review (a median follow-up of 11.1 months) (96).

### Cellular Therapies in PMBCL

CAR-T cell therapy is a cellular therapy that redirects T cells against tumor-associated antigens bypassing the tumor escape mechanism. The success and side effects of this new therapy may depend on the expansion of CAR-T cells in the body. The results of ZUMA-1 (NCT02348216) suggest that CAR-T can induce durable responses and with a median OS of more than 2 years, and has a manageable long-term safety profile in patients with relapsed or refractory DLBCL (97). In NCI trials of autologous anti-CD19 chimeric antigen receptor T cells (anti-CD19 CAR-T) in NHLs, responses to 4 patients with PMBCL included CR (50%), stable disease (25%), and unevaluable (25%), with a duration of response (DOR) of more than 12–22 months (98). Another clinical study showed among the final evaluable patients receiving CAR-T treatment, the achieved DOR percentage over 3 years was 51%, of which DLBCL/PMBCL reached 48% and low-grade lymphoma reached 63%. Finally, the median event-free (EFS) survival of all 45 evaluable patients was 55 months. Except for B cell exhaustion and low blood globulin, long-term adverse reactions were reported to be rare (99). On the basis of this therapy, a digital polymerase chain reaction assay (dPCR) assay was derived to detect transgenic CAR-T cells, which is very conducive to the clinical monitoring of anti-CD19 CAR-T cell therapy (100). As mentioned above, the therapeutic effect of CAR-T is considerable. The FDA has approved CAR-T cell therapy to treat adults with certain types of large B-cell lymphoma (https://www.fda.gov/NewsEvents/Newsroom/PressAnnouncements/ucm581216.htm). The efficacy of CAR-T in PMBCL has not been adequately confirmed and still requires extensive research (101). Although these early clinical trials have included only a small number of patients with PMBCL, these studies have provided a novel therapeutic strategy for the effective treatment of rrPMBCL.

Bispecific antibodies (bs-mAbs) are engineered antibodies presenting two binding sites, in which one binds CD3-positive T cells to CD19-positive B cells, while the other is directed against a co-stimulator on T cells (CD3). This bispecific binding brings B cells and T cells into close proximity, causing T cells to temporarily come into contact with tumor cells (102). The clinical efficacy of blinatumomab (a bispecific CD19-directed CD3 T-cell engager) in the treatment of patients with R/R NHL has been confirmed in various phase I/II trials (103). In the NCT01741792 clinical trial, among 21 evaluable relapsed/refractory diffuse large B-cell lymphoma (rrDLBCL) patients, the total response rate after a single cycle of blinatumomab was 43%, and the CR was 19%. In total, 22% of patients who received progressive dose treatment-experienced Grade 3 neurological events, and 2 patients who received fixed-dose treatment all experienced Grade 3 neurological events (104). Recently, the results of the MT103-104 Phase 1 trial indicated that among the 38 patients subjected to a single-center long-term follow-up analysis, there was no evidence of long-term toxicity, and specifically, there was no evidence of neurocognitive impairment caused by blinatumomab. For the entire study population, the median OS was 4.6 years. In particular, patients who experienced a drug reaction to blinatumomab had a median OS of up to 7.7 years when receiving higher doses (60 mg/m^2^ per day) (103). Epcoritamab is a novel subcutaneously injected bs-mAbs, which has exhibited good safety in phase I/II trials (NCT03625037) and has demonstrated antitumor activity in preliminary studies involving relapsed/refractory B-Non-Hodgkin lymphoma (rrB-NHL). Epcoritamab (GEN3013) is a new bispecific IgG1 antibody that can direct T cells to CD20+ tumor cells. Epcoritamab has been reported to exert strong anti-tumor activity against primary tumor cells present in lymph node biopsies of patients with rrB-NHL, even if this group of patients has received CD20 monoclonal antibody treatment. The results of this study allow us to speculate that epcoritamab may be used for the treatment of newly diagnosed or rrB-NHL patients (105). In summary, studies investigating bs-mAbs in lymphoma mostly involve rrB-NHL. Although there are no specific data reported relative to the application of bs-mAbs in PMBCL, it is undeniable that rrPMBCL presents the typical characteristics of B-NHL. In the future, bs-mAbs may become a novel strategy for rrPMBCL treatment.

## Conclusions and Future Directions

Significant progress has been achieved in the treatment of PMBCL over the last decades. Available studies have shown excellent outcomes with DA-EPOCH-R treatment, which usually allow avoidance of routine mediastinal RT. Alternatively, it is possible to utilize R-CHOP along with end-of-therapy PET-CT evaluation, whose findings with help guide the use of consolidative RT. Nonetheless, the prognosis of rrPMBCL remains dismal. Our primary goal is to improve the treatment regimen of rrPMBCL, to prolong remission and improve outcomes. Recent insight into PMBCL biology has provided the basis for designing treatments that incorporate target agents. In particular, pembrolizumab and CAR-T cell therapy have demonstrated the most promise in PMBCL outcomes. However, it is unlikely that a novel agent could be curative as monotherapy, while a rational combination with chemotherapy drugs might paint a brighter therapeutic perspective.

## Author Contributions

HZh and LX conceived and designed the study and reviewed the manuscript. HC and TP collected, arranged, and wrote the manuscript. YH, RZ, YL, LY, and HZa revised the manuscript. SC and QD designed and prepared the figures and tables. All authors contributed to the article and approved the submitted version.

## Funding

This study was supported by grants from Fundamental Research Funds for the Central Universities of Central South University [No: 2019zzts1002], [No: 2019zzts1060], and [No: 2020zzts785]; the National Natural Science Foundation of China [No.82000200]; Natural Science Foundation of Hunan Provincial Health Commission [No. 20201659]; The Research program of Hunan provincial health and family planning commission [No: B20180496]; "Scientific Research Climbing Plan" of Hunan Cancer Hospital [No. ZX2020003].

## Conflict of Interest

The authors declare that the research was conducted in the absence of any commercial or financial relationships that could be construed as a potential conflict of interest.
